# Cold plasma-induced ovalbumin amyloid fibrils: Morphological characteristics and stability on astaxanthin-loaded high internal phase emulsions

**DOI:** 10.1016/j.fochx.2025.102835

**Published:** 2025-07-24

**Authors:** Chang Liu, Pan-Pan Tang, Xiu-Bin Liu, Najla AlMasoud, Taghrid S. Alomar, Rana Muhammad Aadil, Jun-Hu Cheng, Zhi-Wei Liu

**Affiliations:** a College of Food Science and Technology, Hunan Agricultural University, Changsha 410128, China; b School of Food Science and Engineering, South China University of Technology, Guangzhou 510641, China; c Department of Chemistry, College of Science, Princess Nourah bint Abdulrahman University, PO, Box 84428, Riyadh 11671, Saudi Arabia; d Guangdong Key Laboratory of Food Intelligent Manufacturing, Foshan University, Foshan 528225, China; e Changsha Innovation Institute for Food, Changsha, 410128, China; f National Institute of Food Science and Technology, University of Agriculture, Faisalabad, 38000, Pakistan

**Keywords:** Cold plasma, Ovalbumin, Amyloid fibril, High internal phase emulsion, Astaxanthin

## Abstract

The morphological characteristics of cold plasma (CP)-induced ovalbumin (OVA) amyloid fibrils (OAFs) and their ability to stabilize astaxanthin-loaded high internal phase emulsions (HIPEs) were investigated. The results indicated that CP treatment significantly accelerated OVA fibrillization by inducing its globular structure unfolding and polypeptide cleavage, leading to the rapid formation of short, worm-like fibrils within 10 min of fibrillation. Compared to untreated OVA, which had a diameter of 18.65 ± 6.47 nm, the length of OVA worm-like fibrils progressively increased from 69.17 ± 23.84 nm (10 min) to 114.94 ± 38.04 nm (8 h) before decreasing to 92.24 ± 36.22 nm (24 h). The ability of OVA to stabilize HIPEs was significantly improved following fibrillation. Notably, the shorter fibrils of OAFs-10 min and OAFs-24 h exhibited superior stability in HIPEs compared to the longer fibril of OAFs-8 h. The reason may be ascribed to the robust interface adsorption ability of OAFs-10 min and OAFs-24 h with shorter length, generating a dense and thick interfacial layer in the oil/water emulsion (maximum 11.57 ± 1.98 %), as confirmed by confocal laser scanning microscopy (CLSM) imaging and interface protein adsorption analysis (maximum 31.61 ± 2.49 %), thereby preventing droplet coalescence. Additionally, HIPEs stabilized by OAFs-10 min exhibited a higher oil fraction (82.5 %), superior ionic (1200 mM) and thermal stability (100 °C), and greater astaxanthin retention (91.55 ± 1.97 %, 1200 mM) compared to those stabilized by other OAFs. Meanwhile, HIPEs stabilized by OAFs-24 h exhibited enhanced stability under freeze-thaw cycles and centrifugation, with centrifugal stability constant (Ke) of 16.18 ± 4.78 %.

## Highlights

• The morphological polymorphism of CP-induced OAFs for stabilizing HIPEs was investigated.
• HIPEs stabilized by OAFs-10 min exhibited higher oil fractions, enhanced ionic and thermal stability
• HIPEs stabilized by OAFs-10 min exhibited better astaxanthin retention compared to HIPEs prepared by other OAFs.
• HIPEs encapsulated by OAFs-24 h displayed improved freeze-thaw cycles and centrifugation stability.

## 1 Introduction

Recently, protein amyloid fibrils have garnered significant attention due to their high aspect ratio, outstanding molecular flexibility, and remarkable biocompatibility, resulting in exceptional functional properties such as emulsifying, foaming, and gelling (Pazla et al., 2023; Zhang et al., 2024). Fibrous proteins are primarily composed of stacked cross-β structures along the fibril axis, which are characterized by tightly packed molecular arrangements and stabilized through hydrogen bonds and hydrophobic interactions (Xu, Ma, et al., 2023). Compared to other protein-based polymers, protein amyloid fibrils exhibit distinct advantages in rigidity, chirality, and surface charge properties, highlighting their broad potential in diverse fields such as biomedicine (Abdelrahman et al., 2020), food materials (Zhang et al., 2024), and tissue engineering (Hekmat et al., 2024). Importantly, recent studies have also confirmed that food protein amyloid fibrils pose no significant toxicity risks, supporting their safe use in food applications (Cao & Mezzenga, 2019; Xu, Zhou, et al., 2023). Native proteins have been widely applied in the food industry owing to their abundant availability, nutritional richness, and functional versatility. However, the emulsifying performance of native proteins is often constrained by their limited conformational stability and interfacial activity, particularly under complex processing conditions where denaturation or aggregation may occur, leading to deterioration in functionality. In recent years, the rational modulation of protein self-assembly into amyloid fibrils has progressively been recognized as an appealing strategy to enhance and expand the functional applications of native food proteins (Zhao et al., 2025).

Protein fibrils derived from plant and animal proteins have proven effective in stabilizing emulsions compared to their corresponding precursor proteins, offering a robust alternative to traditional emulsifiers that often fail under harsh conditions (Bai et al., 2021; McClements & Jafari, 2018). Microstructural polymorphism is a defining characteristic of amyloid fibrils, commonly observed even in assemblies formed from identical protein monomers (Xu, Ma, et al., 2023). Additionally, the interfacial adsorption behavior of protein fibrils in emulsions depends on their morphology, length, flexibility, and surface hydrophobicity (Yue et al., 2022). Therefore, many strategies have been developed to modulate the microstructure of protein fibrils for superior emulsifying properties. According to Feng et al. (2024), the emulsifying activity is significantly increased through interactions between soybean protein amyloid fibrils and chitosan. In a study, the authors demonstrated that shorter glycated whey protein fibrils stabilized Pickering emulsions more effectively by promoting better adsorption and aggregation at oil droplet surfaces (Halavach, 2024; Liu et al., 2020). Cui et al. (2022) further showed that electrostatic interactions between fibrils with diverse morphologies and aggregation states could affect emulsion properties. The two typical methods for preparing amyloid fibrils from food proteins involve heating the protein above its denaturation temperature (80–90 °C) at a pH below its isoelectric point or low ionic strength, with magnetic stirring for 5–24 h (Cao & Mezzenga, 2019). Moreover, strategies to accelerate the fibrillization rate of traditional acidic-heating method through non-thermal physical treatment have also been explored, including ultrasound (Kozell et al., 2024), high pressure (Shah et al., 2012), and low-frequency magnetic fields (Liu, Yang, Wang, et al., 2024). For example, Liu, Chen, Cheng, et al. (2024) reported that longer amyloid fibrils of pea globulin (PG) were obtained after being treated with low-frequency magnetic fields compared to native PG, which exhibited superior solubility, emulsifying, and gelling properties. Cold plasma (CP) technology, an emerging physical technique that harnesses reactive oxygen and nitrogen species (RONS) to modify food matrices, demonstrates particular promise in food processing applications due to its environmentally friendly, non-thermal, and chemical residue-free characteristics, especially for protein structural modifications (Basak & Annapure, 2022). Our previous study demonstrated that the fibrillation process of CP-treated OVA can be significantly shortened by reducing the time of the lag phase compared with the traditional acidic-heating method (Liu et al., 2025). Although CP has been shown to improve the fibrillation kinetics of OVA, the functional properties of CP-induced OAFs have not been systematically investigated, particularly their emulsifying properties.

Therefore, this study aims to systematically investigate the effect of morphological changes of CP-induced OAFs during fibrillation times on the ability to stabilize the high internal phase emulsions (HIPEs). To achieve this, the morphology and structure of OAFs were examined using atomic force microscopy (AFM) during the fibrillation process. Meanwhile, structural alterations of OVA were monitored by sodium dodecyl sulfate-polyacrylamide gel electrophoresis (SDS-PAGE) and multispectral analysis. Additionally, the emulsifying properties of OAF-stabilized astaxanthin-loaded HIPEs were evaluated by assessing interfacial protein adsorption, dynamic size distribution, rheological behavior, and confocal laser scanning microscopy (CLSM) analysis. Moreover, the performance of OAF-stabilized HIPEs, including their behavior under various stress conditions, including ionic strength, thermal treatments, freeze-thaw cycles, and centrifugation, as well as astaxanthin retention, was evaluated.

## 2 Materials and methods

### 2.1 Materials and chemicals

OVA (95 %) was purchased from Yuanye Bio-Technology (Shanghai, China). Fast green and 8-anilino-1-naphthalene sulfonic acid (ANS) were obtained from Aladdin Chemicals (Shanghai, China). Thioflavin T (ThT) was sourced from Sigma-Aldrich (MO, USA). Soybean oil, astaxanthin (96 %), and Nile blue were purchased from Macklin (Shanghai, China). All other reagents were of analytical grade.

### 2.2 The preparation of OAFs with CP treatment

The CP-induced OAFs were prepared based on our previous method with slight modifications (Z.-W. Liu et al., 2025). OVA (10 mg/mL, *w/v*) was dissolved in deionized water, and 5 mL of the solution was subjected to 2 min of CP treatment using a DBD plasma device (Nanjing Suman Electronics Co., Ltd., Nanjing, China) at 40 kV and 13 kHz. Following CP treatment, the solution was heated in a water bath at 85 °C for 10 min, 1 h, 8 h, or 24 h. These time points were chosen based on our previous findings (Liu et al., 2025), and were extended to include shorter (10 min) and longer (24 h) intervals to further investigate the dynamic morphological evolution of CP-induced fibrillation. After each heating duration, samples of the liquid were collected for further analysis. Samples were then immediately cooled in an ice bath and subsequently stored at 4 °C until analysis. Untreated OVA samples were adjusted to the same pH as the CP-heated OAF samples to ensure comparability. The OAFs obtained at 10 min, 1 h, 8 h, or 24 h were donated as OAFs-10 min, OAFs-1 h, OAFs-8 h, and OAFs-24 h, respectively.

### 2.3 AFM

The AFM was employed to evaluate the morphology of OAFs (Liu et al., 2025). Samples were diluted to 4 μg/mL in deionized water. A 10 μL aliquot was applied to a freshly cleaved mica disk and dried for 6 h at 25 °C. Fibril images were captured using an AFM instrument (MFP-3D-SA, Asylum Research Inc., USA). Data were analyzed with Gwyddion and FiberApp software (Zhao et al., 2025).

### 2.4 Structural characterizations of OAFs

#### 2.4.1 SDS-PAGE

The protein patterns of CP-induced OAFs were analyzed by SDS-PAGE, following our previous method with slight modifications (Liu, Tang, Liu, et al., 2024). Samples (1 mg/mL) were mixed with reducing buffer (1:4 *w*/*v*) and heated at 90 °C for 10 min. Electrophoresis was performed on FuturePAGE™ 4–20 % Bis-Tris gels with 10× MOPS-SDS buffer (ACE Biotechnology Co., Ltd., Nanjing, China) in a WIX-mulitiPRO2 system (WIX Technology Co., Ltd., Beijing, China) at 160 V for 45 min. Gels were stained with BeyoBlue™ (Beyotime Biotechnology Co., Ltd., Shanghai, China) for 1 h and subsequently destained with deionized water for 2 h. Electrophoresis gel images were captured using a Canon EOS 200D II digital camera.

#### 2.4.2 ThT fluorescent analysis

ThT binding assays for untreated OVA and OAFs were conducted as described previously (Tang et al., 2024). A ThT stock solution (8 mg dissolved in 10 mL of 10 mM phosphate buffer, pH 7.0, containing 150 mM NaCl) was diluted 50 times before use. For the assay, 40 μL of each sample (10 mg/mL) was mixed with 4 mL of the diluted ThT solution. Fluorescence was measured using a Varioskan Flash microplate reader (Varioskan Flash, Thermo Fisher Scientific, Finland) at excitation and emission wavelengths of 440 nm and 480 nm, respectively, with both slits set to 12 nm.

#### 2.4.3 FTIR

FTIR spectra of the untreated OVA and OAF samples were acquired following the procedure described in our earlier work (Liu, Tang, Zhang, et al., 2024). Freeze-dried samples were thoroughly ground and mixed with KBr at a mass ratio of 1:100, and then pressed into a thin slice. Spectra were recorded in the range of 400–4000 cm^−1^ with an IRAffinity-1 spectrometer (Shimadzu, Japan). The resolution was set at 4 cm^−1^ with 32 scans per sample. The amide I region (1600–1700 cm^−1^) was analyzed using PeakFit 4.12 software.

#### 2.4.4 Surface hydrophobicity analyses

The surface hydrophobicity of untreated OVA and OAF samples was evaluated according to the method outlined in our previous study with minor modifications (Liu et al., 2025). Specifically, 5 mL of the sample solution was mixed with 50 μL of ANS solution (5.0 mM, 0.1 mM phosphate buffer). The mixture was then kept in the dark at room temperature for 30 min. Fluorescence measurements were performed on a fluorescence spectrophotometer (F7000, Shimadzu, Kyoto, Japan) with an excitation wavelength of 380 nm and an emission scan range of 400–600 nm. Both excitation and emission slit widths were set to 2.5 nm.

### 2.5 Fibril conversion rate analysis

The fibril conversion rate was determined using a method adapted from Zhao et al. (2025). Samples (2 mg/mL) were placed into 100 kDa ultrafiltration tubes and centrifuged at 5500 ×*g* for 20 min, a step repeated four times. The retained material was washed twice after each centrifugation to remove unaggregated proteins. The protein content in the filtrate was measured using the BCA assay (Walker, 2009). The fibril conversion rate was calculated as follows:

(1) $$\text{Fibril conversion rate}\;\left(\%\right)=\frac{P_{t}-P_{u}}{P_{t}}\times 100$$

Where P_t_ is the total protein mass (mg), and P_u_ is the mass of unaggregated (soluble) protein measured in the filtrate after ultrafiltration (mg).

### 2.6 Preparation of HIPEs loaded with astaxanthin

The preparation of HIPEs encapsulating astaxanthin followed the procedures outlined by Song et al. (2023), with minor modifications. Astaxanthin (20 mg) was dissolved in a 3:1 (*v*/v) acetone/dichloromethane mixture (10 mL) to prepare a 3.34 mM solution, which was then filtered through a 0.22 μm filter. Subsequently, 30 μL of this solution was added to 3.9 mL of soybean oil to achieve a final astaxanthin concentration of 20 μM. HIPEs were prepared by mixing 75 % oil phase and 25 % aqueous phase (10 mg/mL OAFs) and homogenizing at 12360 ×g for 2 min.

### 2.7 Characterizations of HIPEs

#### 2.7.1 Interfacial protein adsorption

The interfacial protein adsorption of HIPEs was quantified based on the method described by Yuan et al. (2024), with slight modifications. HIPEs were centrifuged at 19320 ×*g* for 30 min, and the aqueous phase was subsequently collected. The concentration of unbound protein in the aqueous phase was determined using the BCA assay (Walker, 2009). The interfacial protein adsorption was calculated as follows:

(2) $$\text{Interfacial protein adsorption}\;\left(\%\right)=\frac{C_{0}-C_{f}}{C_{0}}\times 100$$

Where C_0_ is the initial protein concentration and C_f_ is the unabsorbed protein concentration after centrifugation.

#### 2.7.2 Particle size

The mean particle sizes of the HIPEs were measured utilizing a laser diffraction particle size analyzer (MASTERSIZER 3000, Malvern Instruments Ltd., Worcestershire, UK) at 25 °C (Tang et al., 2024). A refractive index of 1.45 was set for the protein phase and 1.33 for the solvent. All measurements were performed in triplicate to ensure data reliability and reproducibility.

#### 2.7.3 Optical microscopy

The microscopic morphology of the HIPEs was observed utilizing an Olympus CX31 microscope (Tokyo, Japan) at 10× magnification (Liu, Yang, Wang, et al., 2024). A 50 μL sample of HIPE mixed 1:1 with 0.1 % SDS was placed on a slide, and images were captured with the microscope's camera.

#### 2.7.4 Rheological properties

The rheological properties of OAF-stabilized HIPEs were evaluated utilizing a rotational rheometer (Kinexus Rotational Rheometer, Malvern Instruments, Malvern, UK) with a 40 mm plate and 1.0 mm gap (Song et al., 2023). Viscosity was measured in the steady shear mode at shear rates of 0.1–100 s^−1^. The storage modulus (G′) and loss modulus (G′′) were determined in the dynamic shear mode (1.0 Hz, 0.01–100 % strain) to identify the linear viscoelastic region (LVR). A frequency sweep was also conducted to recorded G′ and G′′ at 0.1 % strain (within the LVR) over 0.1–10 Hz.

#### 2.7.5 CLSM

The microstructure of the HIPEs was analyzed utilizing a confocal laser scanning microscope (Carl Zeiss Inc., LSM710, Germany), following the method outlined in our previous study (Liu, Chen, Cheng, et al., 2024). Nile red (3 mg) was dissolved in 25 mL soybean oil to prepare the oil phase, which was then emulsified with the protein solution to form HIPEs. A 0.5 mL aliquot of the emulsion (200 μL HIPEs, 132 μL of 0.1 % SDS solution, and 666 μL low-melting-point agarose) was mixed with 30 μL of 0.1 % (*w/v*) Fast Green solution. After thorough mixing, 50 μL of the mixture was transferred onto a glass slide for imaging. Dual-channel fluorescence images were captured using a 40× objective lens with excitation wavelengths of 488 nm (for Nile red-labeled oil phase) and 633 nm (for Fast Green-labeled protein phase), covering a field of view of 1024 μm × 1024 μm.

### 2.8 Stability of astaxanthin-loaded HIPEs prepared by OAFs

#### 2.8.1 Formation of HIPEs with different oil phase ratios

The HIPEs were prepared according to the procedure described in Section 2.6 with oil phase proportions of 75.0, 77.5, 80.0, 82.5, and 85.0 %, respectively.

#### 2.8.2 Ionic stability

The ionic stability of astaxanthin-loaded HIPEs was also evaluated (Li et al., 2022). NaCl was added to the oil-aqueous mixtures to achieve concentrations of 300 and 1200 mM. After preparing HIPEs as described in Section 2.6, their visual appearance, droplet size, microstructure, rheological properties, and astaxanthin retention rate were evaluated.

#### 2.8.3 Thermal stability

The thermal stability of astaxanthin-loaded HIPEs was assessed based on Song et al. (2023), with modifications. A 5 mL sample of HIPEs was placed in capped glass bottles and heated in water baths at 80 °C and 100 °C for 30 min, respectively. After heating, samples were cooled to room temperature in an ice-water bath. The appearance, droplet size, microstructure, and rheological properties of the HIPEs were analyzed, and the astaxanthin retention rate was determined.

#### 2.8.4 Freeze-thaw stability

The freeze-thaw stability of HIPEs was evaluated using the method outlined by Wang et al. (2022), with minor modifications. A 5 mL sample was frozen at −20 °C for 24 h and thawed at 25 °C for 2 h. Visual appearance was recorded after each freeze-thaw cycle. Samples were homogenized at 5500 ×g for 2 min after thawing to evaluate their re-emulsification and self-supporting capability. Droplet size and distribution were analyzed after the third cycle using an Olympus microscope (Olympus, Japan).

#### 2.8.5 Centrifugal stability

The centrifugal stability of HIPEs was also analyzed (Zhao et al., 2025). A 4 mL sample was centrifuged at 5500 ×*g* for 10 min and photographed. The absorbance of the samples (diluted 100 times with deionized water) was measured at 500 nm using a microplate reader (Thermo Fisher, USA) before and after centrifugation. The centrifugal stability constant (Ke) was calculated using the formula:

(2) $$\mathrm{Ke}\;\left(\%\right)=\frac{A_{1}-A_{0}}{A_{1}}\times 100$$

Where A_1_ and A_0_ represent the absorbance of the HIPEs before and after centrifugation.

#### 2.8.6 Astaxanthin retention rate

Astaxanthin was extracted by mixing 1 mL of HIPEs with 6 mL of extraction solvent (dichloromethane/methanol = 2:1, *v*/v) and then centrifuged at 2150 ×*g* for 20 min. The dichloromethane layer was collected, and its absorbance was measured at 489 nm using a microplate reader (Thermo Fisher, USA). Astaxanthin content was determined using the standard curve (Y = 0.2446× - 0.0198, R^2^ = 0.999). Retention rate was calculated as follows:

(3) $$\text{Astaxanthin retention rate}\;\left(\%\right)=\frac{C_{S}}{C_{0}}\times 100$$

Where C_S_ represents the concentration of astaxanthin in the sample, and C_0_ is the initial concentration of astaxanthin.

### 2.9 Statistical analysis

All experiments were performed in triplicate, and all data were presented as the mean ± standard deviation (SD). Data analysis was performed by conducting a one-way analysis of variance (ANOVA) followed by the least significant difference (LSD) post hoc test, conducted with SPSS statistical software. Statistical significance was defined at *P* < 0.05.

## 3 Results and discussion

### 3.1 Morphological alteration of CP-induced OAFs during fibrillation process

**Fig. 1:**
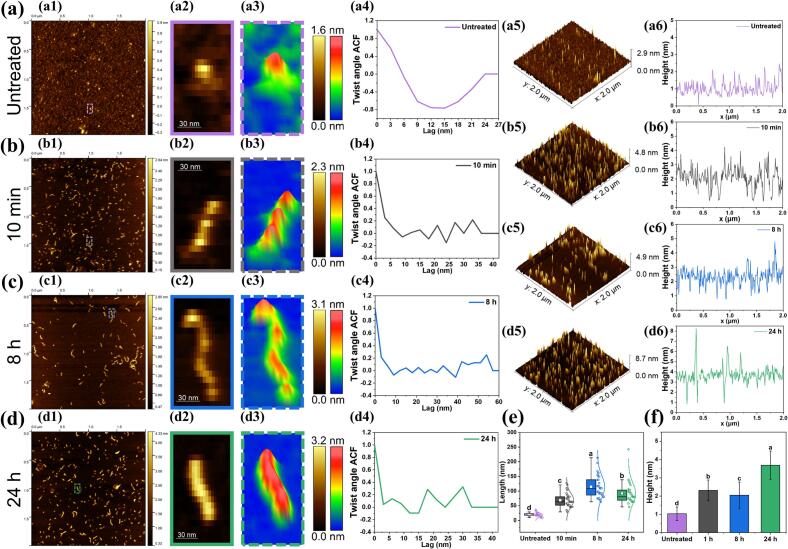
Morphological properties of CP-promoted OAFs. (a1-d1) AFM images; (a2-d2) The interception and amplification of the AFM images in a1-d1 (purple, grey, blue, and green dotted boxes); (a3-d3) Magnified AFM 3D images of target fibrils; (a5-d5) Surface morphology; (a6-d6) The height profiles of the AFM images; (e) The internal contour length distribution; (f) Average height distribution of untreated OVA and CP-promoted OAFs with different heating times (10 min, 8 h, and 24 h). (For interpretation of the references to colour in this figure legend, the reader is referred to the web version of this article.)

The changes in morphology of untreated OVA and OAFs at different stages of the fibrillation process (10 min, 8 h, and 24 h) were examined using AFM. As shown in Fig. 1 a1 and b1, short, worm-like fibrils emerged after 10 min of fibrillation, indicating that CP treatment significantly accelerated the initiation of cross-β structure stacking and the fibrillation of OVA. Upon extending the fibrillation time, the length of OAFs notably increased (Fig. 1 a1-d1 and Fig. 1e). Further morphological analyses using detailed magnifications of typical fibrils (Fig. 1 a2-d2 and Fig. 1a3-d3) revealed that fibril length initially increased, peaking at OAFs-8 h, and then decreased at OAFs-24 h, whereas fibril diameter consistently increased throughout the fibrillation process. Quantitative data corroborated that compared to untreated OVA (diameter: 18.65 ± 6.47 nm), the length of OAFs gradually increased from 69.17 ± 23.84 nm (10 min) to 114.94 ± 38.04 nm (8 h), and subsequently decreased to 92.24 ± 36.22 nm (24 h) (Fig. 1e). This phenomenon indicated that the fibrillation process involved an initial elongation stage followed by a subsequent degradation or dissociation stage (Chen et al., 2022; Zhang et al., 2021). Meanwhile, the diameter of fibril steadily increased from 1.03 ± 0.37 nm (untreated) to 3.69 ± 0.77 nm (OAFs-24 h) (Fig. 1a5-d5, 2a6-d6, and 2f). Similarly, Nicoud et al. (2015) reported that high temperature and agitation could cause amyloid fibrils to fragment but increase in surface area or height as fragmented pieces aggregate. This can be attributed to the temperature sensitivity of the non-covalent interactions, such as hydrogen bonding and hydrophobic interactions, that stabilize fibrils. As the heating was prolonged, these interactions are weakened, resulting in the β-sheet stacks within the fibrils to partially dissociate (Chen et al., 2022). Additionally, Fig. 1 a4-d4 demonstrates variations in the twist angle of amyloid fibrils with fibrillation durations, showing rapid changes for OAFs-10 min and stabilized patterns for the samples fibrillated for 8 h and 24 h. This suggests a transition from highly variable to more stabilized fibril structures as heating duration increases (Zhao et al., 2025). In summary, AFM analysis demonstrated that CP treatment could effectively initiate fibril nucleation and accelerate the rate of fibril formation. Extended fibrillation time led to fibril elongation, while further prolonged fibrillation resulted in fibril fragmentation into shorter, more compact structures. These findings unveiled the dynamic change in the morphology of CP-induced OAFs during the fibrillation process.

### 3.2 Structural analyses of OVA during fibrillation process

**Fig. 2:**
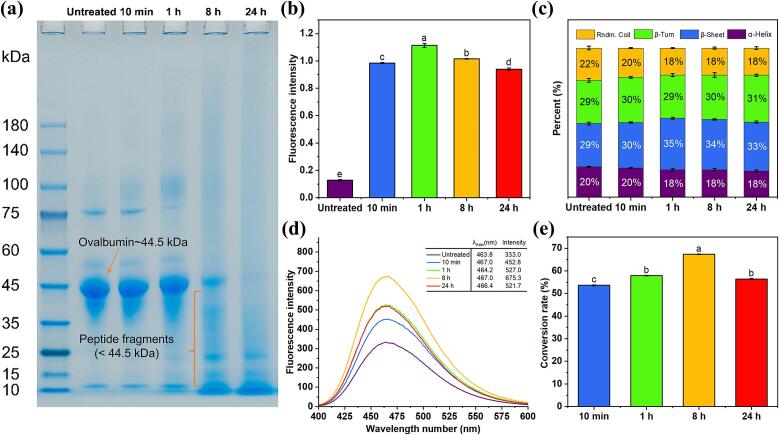
The structural analyses of untreated OVA and CP-promoted OAFs. (a) The protein pattern under the reducing condition; (b) ThT fluorescent intensity; (c) The content of secondary structure; (d) Surface hydrophobicity of untreated OVA and CP-promoted OAF with different heating times. (e) The conversion rate of the fibrils.

Of note, partial unfolding is an obligatory step for globular food proteins to expose fibril-prone regions that are buried within their sphere-like structures (Cao & Mezzenga, 2019). This process involves the unfolding of the globular structure, hydrolysis of polypeptide chains, release of fibril-prone segments, adoption to β-conformation (building blocks), and self-assembling into cross-β fibril spin structures (Liu et al., 2025; Xu, Zhou, et al., 2023). As shown in Fig. 2a, the band corresponding to untreated OVA (∼44.5 kDa) gradually faded and almost disappeared after 8 h of heating, accompanied by the generation of smaller peptide fragments (< 44.5 kDa). This observation indicated that the polypeptide chain of OVA was cleaved, and polypeptides were generated. The self-assembly and reassembly of polypeptides into cross-β motifs were confirmed by ThT analysis, which is considered the “gold standard” for detecting amyloid fibrils formation (Frey et al., 2024). As depicted in Fig. 2b, the untreated OVA exhibited the lowest ThT value of 0.13 ± 0.01. After 10 min of heating, the ThT value of CP-treated OAFs increased rapidly to 0.98 ± 0.01, suggesting rapid nucleation and elongation of fibrils with no apparent lag phase, consistent with our previous findings (Liu et al., 2025). The ThT value peaked at 1.11 ± 0.01 after 1 h, indicating substantial formation of β-sheet structures. This rapid increase suggests significant amyloid fibril formation shortly after CP-heating treatment. In contrast, the traditional acidic-heating method showed much slower kinetics, with ThT fluorescence remaining below 0.25 across all heating durations (Liu et al., 2025), underscoring the efficiency of CP treatment in promoting fibrillation. This finding was supported by AFM analysis in Fig. 1. These results were further verified by FTIR spectroscopy, which analyzed the secondary structural alterations of OVA during the fibrillation process. As shown in Fig. 2c, the β-sheet content significantly increased from the initial 29.07 ± 0.97 % (untreated) to 30.42 ± 0.38 % (OAFs-10 min), 34.62 ± 0.61 % (OAFs-1 h), 33.74 ± 0.42 % (OAFs-8 h), and 32.82 ± 0.76 % (OAFs-24 h) during fibrillation. These results confirmed that the conformational transition of hydrolyzed peptides to β-strands facilitated the formation of cross-β motifs and subsequent self-assembly into protein fibrils.

Surface hydrophobicity analysis revealed the structural changes and driving forces in CP-induced OAF formation (Fig. 2d). The fluorescence intensity of ANS initially increased from 333.0 (untreated) to 675.3 (OAFs-8 h), before decreasing to 521.7 (OAFs-24 h). This trend indicated the unfolding of OVA's native structure, leading to the exposure of buried hydrophobic amino acids, which are crucial for early amyloid fibril formation. During the fibrillation process, cross-β structures self-assembled through hydrophobic interactions, leading to a decline in surface hydrophobicity as exposed groups became buried in the fibril core (Z. Xu et al., 2022). These interactions played a crucial role in the formation of highly organized fibrillar structures in CP-induced OAFs. Similar trends in soy protein and arachin amyloid fibrils further underscore the importance of hydrophobic interactions in the assembly and stacking of cross-β-sheet structures (Yang et al., 2023; Zhao et al., 2025). The conversion rate analysis of OAFs revealed that 53.64 ± 0.19 % protein fibrils formed after 10 min of fibrillation (Fig. 2e), which was consistent with ThT analysis (Fig. 2b), demonstrating CP's effectiveness in accelerating amyloid fibril formation. This rate increased to a peak of 67.33 ± 0.13 % in OAFs-8 h before declining to 56.40 ± 0.18 % in OAFs-24 h, likely due to fragmentation and structural reorganization (Wang et al., 2020; Zhang et al., 2021). ·Taken together, these findings on structural alteration of OVA aligned well with the morphological changes observed in OAFs during the fibrillation process.

### 3.3 Characterization of astaxanthin-loaded HIPEs stabilized by CP-induced OAFs

#### 3.3.1 Characterization of HIPEs

**Fig. 3:**
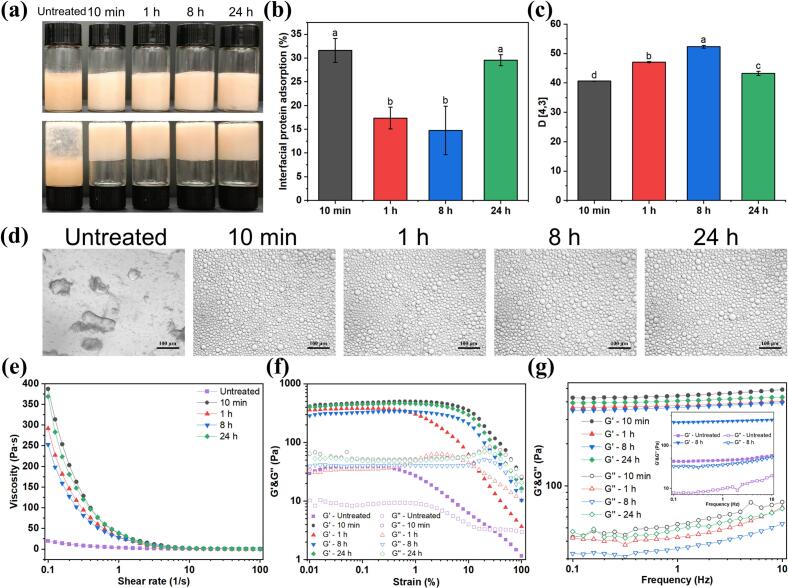
Effect of OAFs on the properties of HIPEs loaded with astaxanthin. (a) Visual appearance; (b) The interfacial protein adsorption; (d) Optical micrographs, scale bar is 100 μm. Rheological properties of OAFs stabilized HIPEs: (e) Viscosity; (f) Strain; (g) Frequency.

**Fig. 4:**
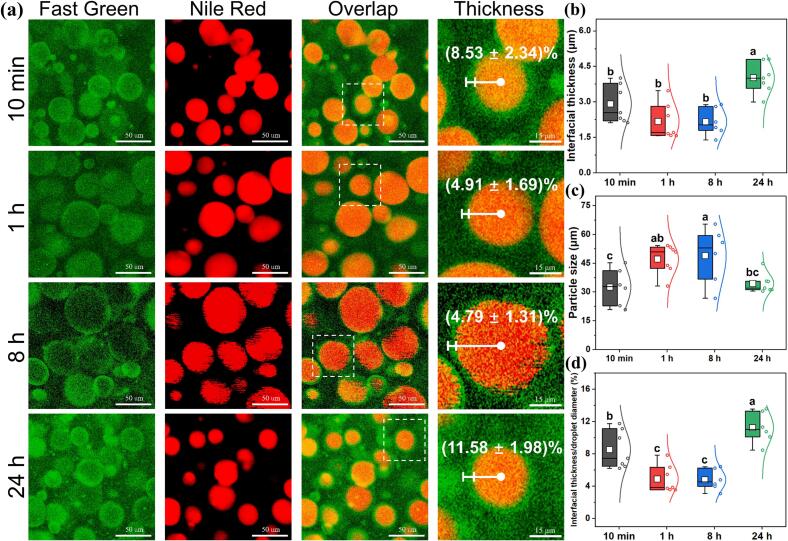
CLSM images OAFs stabilized HIPEs. (a) CLSM images and the magnified interfacial thickness of the droplets (white dotted boxes), oil droplets were dyed by fast green and the proteins were dyed by Nile red. (b) The interfacial thickness; (c) Particle size distribution; (d) Interfacial thickness/droplet diameter of the HIPEs. (For interpretation of the references to colour in this figure legend, the reader is referred to the web version of this article.)

To evaluate the characteristics of OAF-stabilized HIPEs, interfacial protein adsorption, particle size, and microscopic morphology analyses were conducted. As shown in Fig. 3a, greater capacity to stabilize the HIPEs (75 % oil phase) was exhibited by CP-induced OAFs, except for untreated OVA. The proportions of proteins adsorbed at the interface were quantified to assess the HIPE stabilizing capability of OAFs. Normally, increased protein adsorption at the oil-water interface leads to a thicker protein layer, reducing droplet coalescence (Yuan et al., 2024). Notably, the HIPE prepared by OAFs-10 min exhibited maximum interfacial protein adsorption of 31.61 ± 2.49 %, followed by adsorption percentages of 29.56 ± 1.14 % (OAFs-24 h), 17.34 ± 2.29 % (OAFs-1 h), and 14.75 ± 5.13 % (OAFs-8 h) (Fig. 3b). These results were directly correlated to the length of OAFs (Fig. 1), which demonstrated that short fibrils (OAFs-10 min and OAFs-24 h) exhibit higher interfacial capacity than long fibrils (OAFs-1 h and OAFs-8 h). These findings were further validated by subsequent CLSM results (Fig. 4). Consequently, higher adsorption led to greater protein loading at the oil-water interface (Nie et al., 2023), facilitating the formation of a thicker protein layer around oil droplets, thereby improving emulsion stability (Yuan et al., 2024). The stronger interfacial interaction of shorter fibrils (OAFs-10 min and OAFs-24 h) promoted the formation of a more robust protective layer around the droplets (Y. Liu et al., 2024). Furthermore, the particle size distribution of OAF-stabilized HIPEs reflected the emulsifying properties of CP-induced OAFs (Fig. 3c). Specifically, the diameters were 42.5 ± 1.2 μm (OAFs-10 min), 48.7 ± 1.5 μm (OAFs-1 h), 52.3 ± 1.8 μm (OAFs-8 h), and 45.6 ± 1.4 μm (OAFs-24 h). These sizes exhibited an inverse relationship with interfacial protein adsorption, indicating that higher protein loading at the oil-water interface resulted in smaller droplet sizes and improved emulsion stability (Zhao et al., 2025). Optical microscope images (Fig. 3d) also highlight the overall structural differences between OAF- and OVA-stabilized emulsions, particularly the improved emulsifying properties of OAFs.

#### 3.3.2 Rheological properties of HIPEs

The rheological properties of OAF-stabilized HIPEs were evaluated by measuring their dynamic viscoelasticity and macroscopic properties under varying strains and frequencies. As shown in Fig. 3e, the initial apparent viscosity of the emulsion prepared by untreated OVA was below 50 Pa·s. While the apparent viscosity of HIPEs prepared by OAFs was extremely increased, with the sequence of OAFs-10 min > OAFs-24 h > OAFs-1 h > OAFs-8 h. With increasing shear rate, HIPEs encapsulated by OAFs exhibited clear shear thinning behavior, demonstrating a significant reduction in apparent viscosity. This phenomenon is attributed to the deformation and realignment of HIPE droplets under shear forces (Castel et al., 2017). Stress sweep tests were further performed to assess the viscoelastic properties and identify the LVR of the HIPEs, where the storage modulus (G′) reflects elasticity, and the loss modulus (G″) indicates viscosity. As displayed in Fig. 3f, within the LVR, the emulsions displayed solid-like characteristics, with G′ consistently greater than G″ (W.-J. Liu et al., 2021). As the strain increased beyond the LVR, both G′ and G″ decreased, with G′ eventually intersecting G″, indicating a transition from solid-like to liquid-dominant behavior due to structural breakdown (He et al., 2024). Notably, the values of G′ and G″ for OAF-stabilized HIPEs were significantly higher than those of the untreated OVA mixture, highlighting their superior viscoelastic properties. Among the tested HIPEs, the LVR of HIPEs stabilized by OAFs-1 h was shorter, indicating lower resistance to deformation. Conversely, a broader LVR in other HIPEs suggests greater resistance to deformation (Q. Li et al., 2020). To further examine the dynamic mechanical properties of HIPEs, frequency sweep tests were performed at a constant strain (0.1 %) within the LVR (Fig. 3g). Across the measured frequency range (0.1–10 Hz), G′ consistently exceeded G″, indicating gel-like behavior. Moreover, the G′ and G″ values of OAF-stabilized HIPEs were significantly higher than those of the mixture containing untreated OVA. Specifically, the order of G′ and G″ values from highest to lowest among the tested HIPEs was: OAFs-10 min, OAFs-24 h, OAFs-1 h, and OAFs-8 h. This observation could be associated with the particle size distribution (Fig. 3c) since smaller droplets led to tighter packing, reduced fluidity, and increased resistance to deformation (W. Xu et al., 2021). In summary, HIPEs stabilized by short fibrils (OAFs-10 min and OAFs-24 h) display superior rheological properties compared to those stabilized by long fibrils (OAFs-1 h and OAFs-8 h), owing to the superior mobility and interface adsorption of short fibrils due to their flexibility The results demonstrated that HIPEs stabilized by OAFs-10 min and OAFs-24 h exhibited superior rheological properties due to their higher flexibility and interfacial adsorption efficiency (Y. Liu et al., 2024).

#### 3.3.3 CLSM

The microscopic structure of HIPEs was examined using CLSM (Fig. 4a). Oil droplets were stained with Nile red, and proteins were labeled green. The overlap images showed that oil droplets were encapsulated within proteins, indicating a characteristic oil-in-water emulsion structure. CLSM imaging revealed that emulsions stabilized by OAFs-10 min and OAFs-24 h exhibited smaller droplet sizes and a significant increase in fluorescence intensity at the droplet edges. To quantify changes in individual droplets, the interface layer thickness and particle diameters were measured using ImageJ software and plotted in data distribution maps (Fig. 4b-d). The interface layer formed by OAFs-24 h was the most pronounced, nearly twice as thick as that of OAFs-1 h, followed by OAFs-10 min (Fig. 4b). These findings suggest a strong interface adsorption capacity of OAFs with shorter fibril lengths (OAFs-10 min and OAFs-24 h), resulting in a dense, elastic, and thick interfacial layer in HIPEs (Zhao et al., 2025). Interestingly, HIPEs with thicker interface layers had smaller droplet sizes (Fig. 4c), likely due to enhanced steric hindrance preventing droplet aggregation (Yue et al., 2022). Additionally, the average interfacial thickness to droplet diameter ratio for the HIPEs stabilized by OAFs-24 h reached 11.57 ± 1.98 % (Fig. 4d), while the ratios for the other HIPEs were 8.53 ± 2.34 % (OAFs-10 min), 4.91 ± 1.69 % (OAFs-1 h), and 4.79 ± 1.31 % (OAFs-8 h), respectively. These findings highlight the crucial role of the interfacial layer as a protective barrier against droplet coalescence and emphasize the substantial contribution of CP-induced OAFs to enhancing emulsion stability, particularly due to the superior stabilization provided by shorter fibrils.

### 3.4 Proposed mechanism of CP-induced OAFs in stabilizing HIPEs

**Fig. 5:**
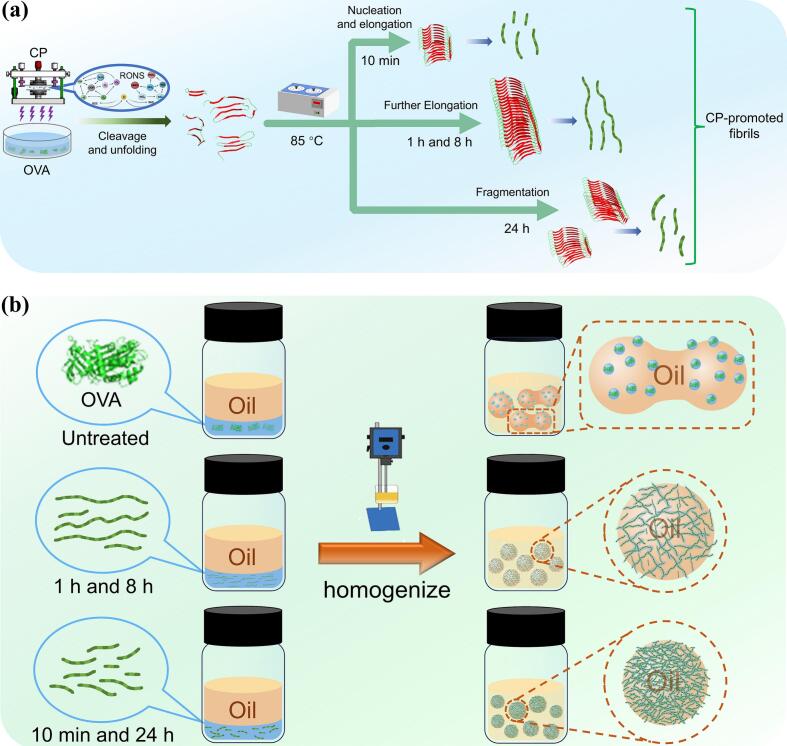
Schematic illustration of the mechanism of (a) the formation of CP-promoted OAFs; (b) HIPEs stabilized by OAFs.

The underlying mechanism for the enhanced stabilization performance of HIPEs by CP-induced OAFs is illustrated in Fig. 5. In the untreated sample, native OVA maintained a compact globular structure that restricted the exposure of its hydrophobic regions, thereby limiting its ability to adsorb at the oil-water interface and resulting in unstable emulsions (Fig. 3) (Tang et al., 2024). Compared with untreated OVA, the CP-induced OAFs underwent structural unfolding and fibrillar stacking during fibrillation, accompanied by multiple structural transformations: (1) The unfolding of OVA (Fig. 2) exposed previously hidden hydrophobic regions, as evidenced by the increased surface hydrophobicity (Fig. 2d) and changes in secondary structure (Fig. 2c). These hydrophobic areas can interact with oil droplets, thereby enhancing the protein's ability to stabilize the oil-water interface in HIPEs (Huyst et al., 2021). (2) The unfolded and cleaved OVA (Fig. 5a) underwent nucleation and elongation to form amyloid fibrils, which can serve as a network structure that restricts droplet nobility and inhibits coalescence (Fig. 5b) due to their high aspect ratio (Chen et al., 2022).

Furthermore, it is noteworthy that the different morphologies of OAFs induced by CP treatment fundamentally contribute to the improved stability of HIPEs, particularly in terms of average lengths. OAFs formed after short (10 min) and prolonged (24 h) heating durations consisted mostly of short fibrils, while those formed after intermediate heating durations (1 and 8 h) were predominantly composed of longer fibrils (Fig. 5a). This morphological difference correlates directly with their interfacial behavior: shorter fibrils exhibit better mobility, enabling more efficient adsorption at the oil-water interface compared to longer fibrils (Fig. 5b). The more flexible and anisotropic properties of the short worm-like fibrils make them more effective at forming dense and elastic interfacial layers, thereby preventing droplet flocculation and improving overall emulsion integrity (G. Liu et al., 2020; Y. Liu et al., 2024). These conclusions are consistently supported by interfacial protein adsorption data (Fig. 3b) and CLSM analyses (Fig. 4), where short fibril samples (OAFs-10 min and OAFs-24 h) showed enhanced protein adsorption and thicker interfacial layers around droplets. Hence, the integrated structural and functional results demonstrate that CP-induced fibrillation of OVA could significantly enhance the emulsifying properties of OVA in stabilizing HIPEs, with the most pronounced improvement observed after 10 min of heating.

### 3.5 Stability of astaxanthin-loaded HIPEs stabilized by OAFs

#### 3.5.1 Stabilization of HIPEs by OAFs with different oil fractions

**Fig. 6:**
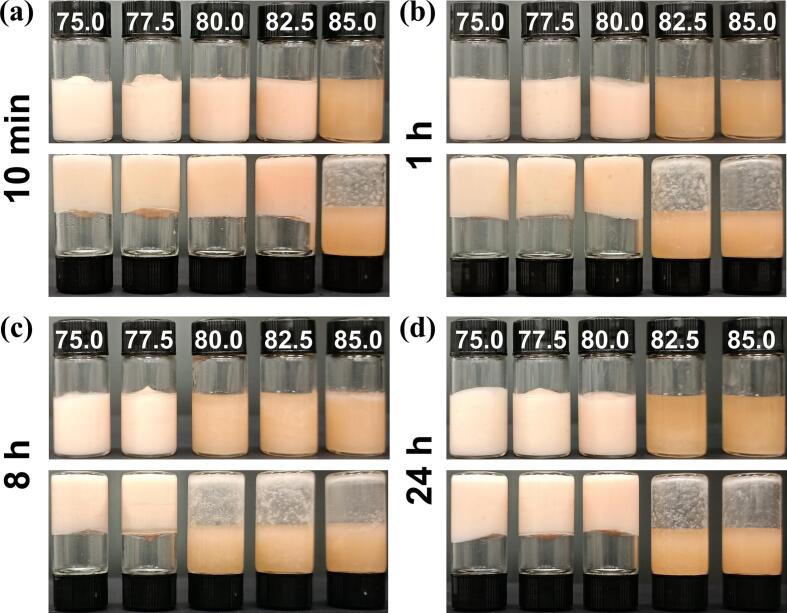
Ability of OAFs to form HIPEs with different oil volume fractions (75–85 %). HIPEs stabilized by OAFs with (a) 10 min, (b) 1 h, (c) 8 h, and (d) 24 h heating.

The oil phase volume fraction is indicative of the stabilization capacity of OAFs in HIPEs systems (Du et al., 2024). In this study, the stabilization capacity of CP-induced OAFs across a range of oil volume fractions from 75.0 % to 85.0 % was evaluated (Fig. 6). Notably, OAFs demonstrated a superior stabilization capacity, effectively supporting oil phases up to 82.5 % (OAFs-10 min), as evidenced by inverted bottle tests (Fig. 6a). In comparison, other OAFs exhibited different maximum stabilization thresholds at 80.0 % (OAFs-1 h), 77.5 % (OAFs-8 h), and 80.0 % (OAFs-24 h) oil phases (Fig. 6b-6d). These findings are consistent with previous reports, such as by Li et al. (2022), where pea protein isolate nanoparticles were shown to be capable of stabilizing HIPEs with an 80 % oil fraction. This highlights the strong potential of CP-induced OAFs as effective stabilizers for HIPEs in applications requiring high oil content emulsions.

#### 3.5.2 Ionic stability

**Fig. 7:**
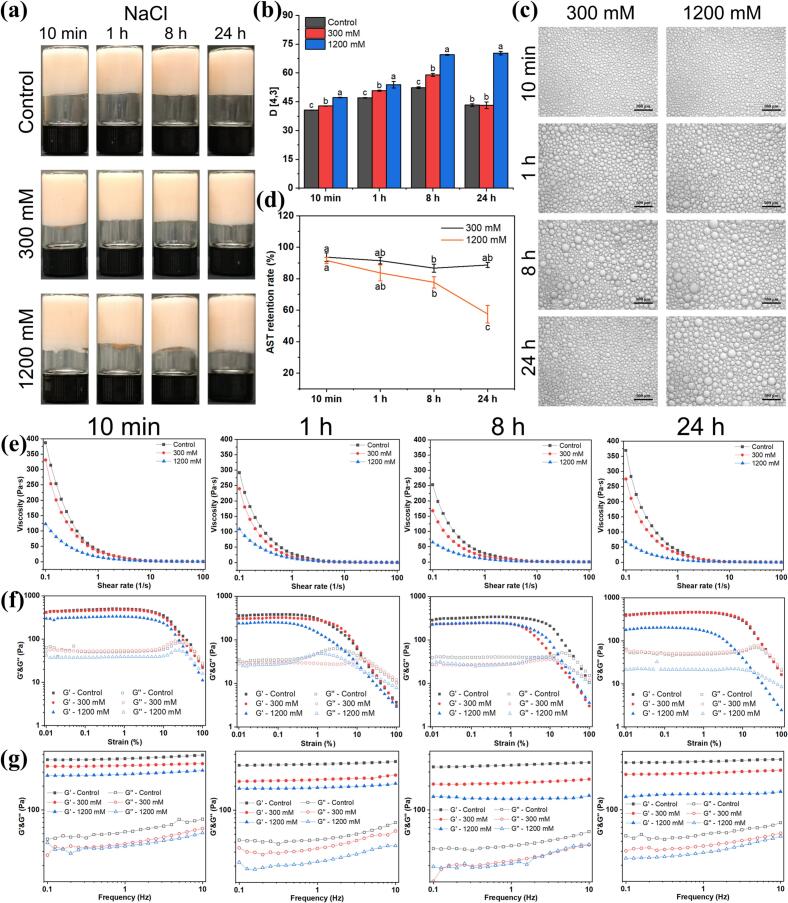
The ionic stability of OAF-stabilized HIPEs. (a) Appearance; (b) Particle sizes; (c) Optical microscope images; (d) Astaxanthin retention rates of HIPEs with NaCl addition. Rheological properties of the HIPEs: (e) Viscosity; (f) Strain; (g) Frequency.

The impact of varying ionic strengths (300 mM and 1200 mM NaCl) on the properties of OAF-stabilized HIPEs were investigated (Fig. 7). Visually, no phase separation was observed, and the HIPEs maintained a gel-like appearance across all tested ionic strengths (Fig. 7a). Microscopic images confirmed that droplets remained densely packed and closely attached under varying ionic conditions (Fig. 7c). Electrostatic repulsion is a critical factor in emulsion stability, but electrostatic screening at high ionic strengths reduces this stabilizing force (X.-M. Li et al., 2019). These findings suggest that steric repulsion also played a role in OAF-stabilized HIPEs under disturbed electrostatic interactions (X.-L. Li et al., 2022). The average droplet size of HIPEs increased with increasing ionic strength. For HIPEs stabilized by OAFs-10 min, the mean droplet size increased from 40.60 ± 0.08 μm (control) to 42.77 ± 0.05 μm (300 mM) and to 47.17 ± 0.09 μm (1200 mM) (Fig. 7b). However, OAFs-24 h-stabilized HIPEs showed a more significant increase in particle size, from 43.23 ± 0.68 μm (control) to 70.30 ± 0.93 μm (1200 mM), indicating coalescence and aggregation due to reduced electrostatic repulsion.

Dynamic rheological analysis revealed a general decline in viscosity apparent of the emulsions with increasing NaCl concentration (Fig. 7e), with the ruptured emulsions showing initial viscosity less than 50 Pa·s. The G′ consistently exceeded G″ in strain sweep across all ionic conditions, indicating gel-like behavior and good stability (Fig. 7f). Both G′ and G″ values declined during strain and frequency sweeps after NaCl addition (Fig. 7f and g), consistent with the changes in particle size (Fig. 7b). Notably, HIPEs stabilized by OAFs-10 min exhibited higher viscosity and greater G′ and G″ values compared to other groups, suggesting superior stability under high ionic strength conditions. Astaxanthin encapsulated in OAF-stabilized HIPEs exhibited superior stability with NaCl (Fig. 7d). The retention rate of astaxanthin in OAF-stabilized HIPEs encapsulated by OAFs-10 min remained notably high, reaching 91.55 ± 1.97 % even at 1200 mM NaCl. Across all treatment groups, retention rates exceeded 77.70 ± 3.65 %, except for the OAFs-24 h group with 57.52 ± 5.61 % retention at 1200 mM NaCl. Overall, these findings underscore that OAFs-10 min with shorter fibril length exhibit superior stability and high astaxanthin retention in response to ionic strength disturbance.

#### 3.5.3 Thermal stability

**Fig. 8:**
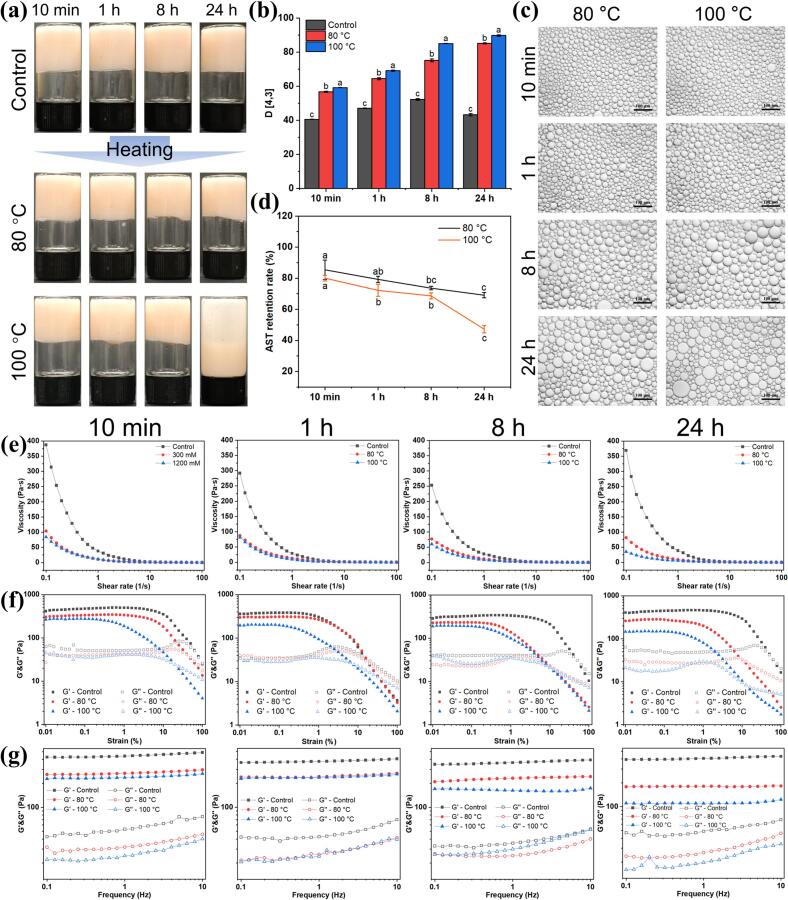
The thermal stability of OAF-stabilized HIPEs. (a) Appearance; (b) Particle sizes; (c) Optical microscope images; (d) Astaxanthin retention rates of HIPEs after heating treatments. Rheological properties of the HIPEs: (e) Viscosity; (f) Strain; (g) Frequency.

Thermal treatment is a common practice in food processing to ensure microbial safety and extend shelf life (Sun et al., 2024). The thermal stability of astaxanthin-loaded HIPEs stabilized by OAFs was evaluated at 80 and 100 °C for 30 min, respectively. At 80 °C, all OAF-stabilized HIPEs maintained their structural integrity and self-supporting ability, without exhibiting phase separation or structural failure (Fig. 8a). These emulsions remained stable upon inversion with no evidence of slippage, indicating robust thermal stability. At 100 °C, only HIPEs stabilized by OAFs-24 h showed signs of rupture, while others retained stability. Particle size analysis (Fig. 8b) and optical micrographs (Fig. 8c) revealed a substantial increase in droplet size after thermal treatment. The mean droplet size of - HIPEs stabilized by OAFs-24 h increased from 43.23 ± 0.68 μm (control) to 89.77 ± 0.45 μm (100 °C), indicating coalescence and aggregation. Despite these changes, the OAF-stabilized HIPEs retained their microstructural integrity even after exposure to elevated temperatures.

Dynamic rheological analyses indicated that the apparent viscosity of the emulsions generally decreased with increased temperature (Fig. 8e), while the ruptured emulsions showed initial viscosity below 50 Pa·s. The G′ and G″ values also declined during strain and frequency sweeps after thermal treatment (Fig. 8f and g), consistent with the observed changes in particle size (Fig. 8b). Notably, HIPEs stabilized by OAFs-10 min exhibited higher viscosity and greater G′ and G″ values compared to other samples, suggesting superior thermal stability. The retention of astaxanthin loaded in HIPEs prepared by OAFs-10 min remained high with the rate of 85.43 ± 6.35 % (80 °C) and 80.05 ± 1.86 % (100 °C). Retention rates exceeded 68.65 ± 1.92 % for all samples except the HIPEs encapsulated by OAFs-24 h (47.22 ± 2.41 %) after 100 °C thermal treatment (Fig. 8d). Similar to the result of ionic stability, OAFs-10 min with shorter fibril length exhibited superior thermal stability and higher astaxanthin retention compared to other OAFs.

#### 3.5.4 Freeze-thaw stability

The freeze-thaw stability of the HIPEs was evaluated through three successive freeze-thaw cycles. As shown in S Fig. 1a, all HIPEs experienced demulsification during these cycles, similar to observations for HIPEs stabilized by gelatin (Mao et al., 2022) and soy protein (Tan et al., 2025). Despite initial susceptibility to freeze-thaw treatment, all HIPEs could be re-emulsified and maintain good self-supporting ability after the first cycles. After the second freeze-thaw cycle, HIPEs stabilized by OAFs-10 min lost their self-supporting ability and slid off when inverted after re-homogenization. By the third cycle, both OAFs-10 min and OAFs-1 h-stabilized HIPEs showed similar behavior, losing their structural integrity. Conversely, HIPEs stabilized by OAFs-8 h and OAFs-24 h retained their self-supporting structures after re-emulsification. These findings were further confirmed by microscopic images of the HIPEs after the third freeze-thaw cycle (S Fig. 1b).

#### 3.5.5 Centrifugal stability

Centrifugation was employed to evaluate the anti-flocculation properties and stability of HIPEs (Zhao et al., 2025). As shown in S Fig. 1c, the emulsions were separated into three distinct layers: an exuded oil phase at the top, an undisturbed emulsion phase in the middle, and an aqueous phase at the bottom. The volume of the exuded oil phase was largest for the mixture with untreated OVA, whereas it was markedly reduced for HIPEs stabilized by OAFs. Specifically, only a negligible oil phase was observed in the HIPEs stabilized by OAFs-8 h and OAFs-24 h. These results were confirmed by the Ke value of HIPEs, an indicator to quantify centrifugal stability, where a lower Ke value indicates higher physical stability and better dispersion of droplets within the emulsion During centrifugation (Zhao et al., 2025). In the current study, the Ke values decreased from 74.93 ± 0.66 % (untreated OVA mixture) to a minimum of 16.18 ± 4.78 % (OAFs-24 h HIPEs). The Ke values for other HIPEs were also significantly lower than that of untreated OVA: 35.20 ± 3.60 % (OAFs-10 min), 54.63 ± 2.21 % (OAFs-1 h), and 24.96 ± 0.91 % (OAFs-8 h) (S Fig. 1d). These results highlight the superior physical stability of HIPEs stabilized with OAFs, with respect to flocculation resistance and phase stratification (Zhao et al., 2025). Analogously, Zhao et al. (2025) also reported that the Ke of emulsions decreased from 89.77 % (SPI) to a minimum of 64.62 % with the stabilization of SPI amyloid fibrils after 12 h acidic-heating treatment.

## 4 Conclusion

In this study, we investigated the morphological polymorphism of CP-induced OAFs for stabilizing HIPEs, focusing on the relationship between their morphology and emulsifying properties. The results revealed that CP treatment significantly accelerated the fibrillation process, leading to the formation of short fibrils within just 10 min of heating, which demonstrated superior emulsifying capacity compared to untreated OVA. The morphology of the fibrils played a crucial role in emulsification efficiency, with shorter fibrils showing superior emulsifying ability, as evidenced by thicker interfacial layers and higher protein adsorption rates compared to longer fibrils. The short fibrils provided a more robust and stable interfacial structure, while longer fibrils contributed more rigid support (Zhao et al., 2025). Additionally, HIPEs stabilized by OAF-10 min exhibited higher oil fractions, enhanced ionic and thermal stability, and better astaxanthin retention compared to HIPEs prepared by other OAFs. While HIPEs encapsulated by OAFs-24 h displayed improved freeze-thaw cycles and centrifugation stability. In conclusion, CP treatment proved to be an effective method for generating OAFs with superior emulsifying properties, highlighting their promise as stabilizers for HIPEs. Further research to explore the application of the CP-induced OAFs-stabilized HIPEs for targeted delivery of bioactive compounds in vivo, should be conducted.

## CRediT authorship contribution statement

**Chang Liu:** Writing – original draft, Formal analysis, Data curation. **Pan-Pan Tang:** Writing – review & editing, Formal analysis. **Xiu-Bin Liu:** Writing – review & editing, Methodology, Formal analysis. **Najla AlMasoud:** Writing – review & editing. **Taghrid S. Alomar:** Writing – review & editing. **Rana Muhammad Aadil:** Writing – review & editing, Validation, Supervision. **Jun-Hu Cheng:** Writing – review & editing, Supervision, Methodology, Investigation, Data curation, Conceptualization. **Zhi-Wei Liu:** Writing – review & editing, Supervision, Software, Methodology, Investigation, Data curation, Conceptualization.

## Declaration of competing interest

The authors declare that they have no known competing financial interests or personal relationships that could have appeared to influence the work reported in this paper.

## Data Availability

Data will be made available on request.

## Appendix A Supplementary data

Supplementary material Image 1
