# Supplementary material for: Cold plasma-induced ovalbumin amyloid fibrils: Morphological characteristics and stability on astaxanthin-loaded high internal phase emulsions
Source: Food Chem X. 2025 Jul 24;29:102835. doi: 10.1016/j.fochx.2025.102835 (PMC12357059; doi:10.1016/j.fochx.2025.102835)
Supplement: Supplementary file 1 — Supplementary material [file mmc1.docx]

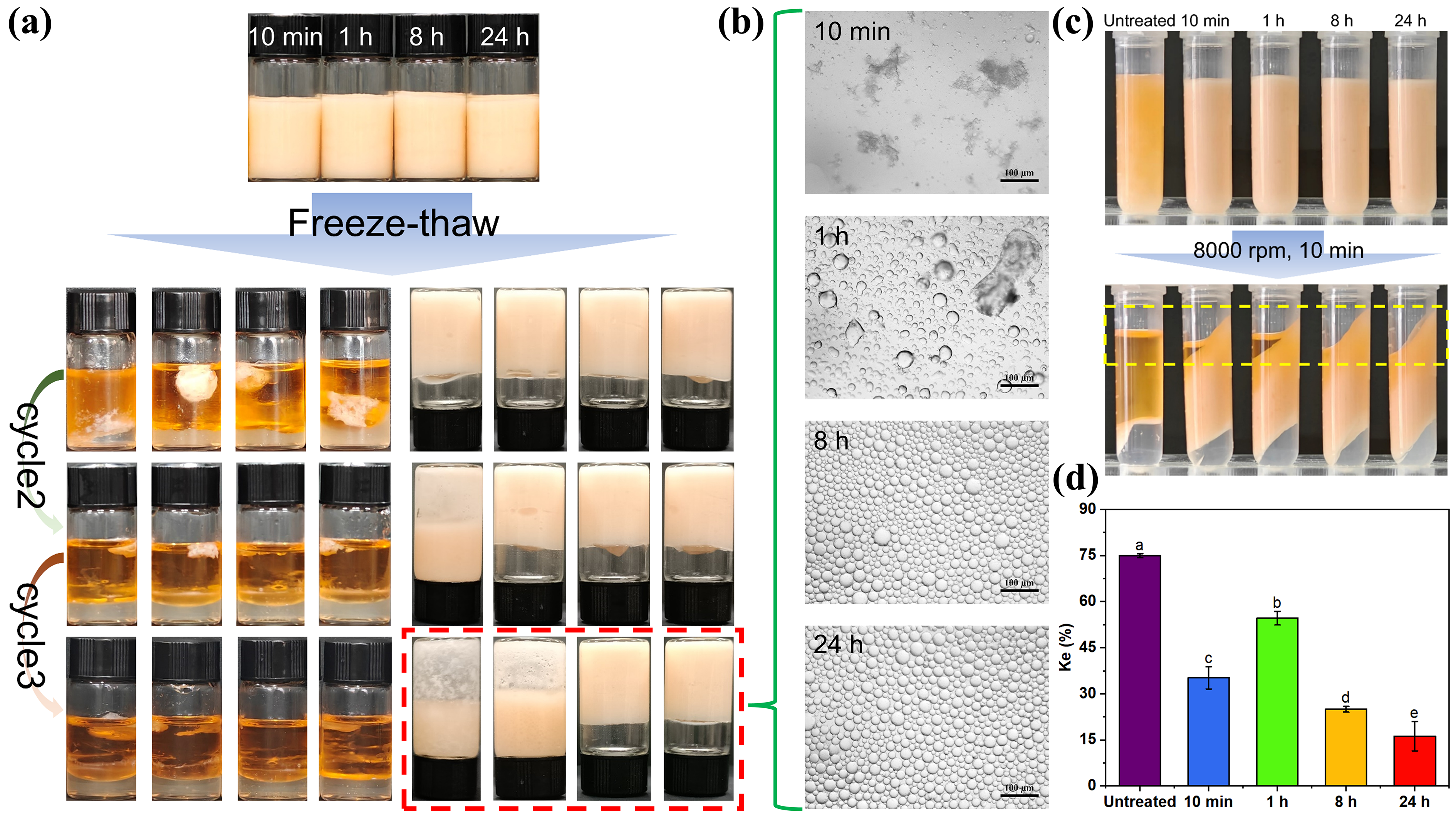


S Fig. 1. The freeze-thaw and centrifugal stabilities of OAF-stabilized HIPEs. (a) Appearance after freeze-thaw treatments; (b) Optical microscope images after the third freeze-thaw cycle; (c) Appearance after centrifugation treatment; (d) The centrifugal stability constant (Ke) of HIPEs.
